# NF-κB contributes to MMP1 expression in breast cancer spheroids causing paracrine PAR1 activation and disintegrations in the lymph endothelial barrier *in vitro*

**DOI:** 10.18632/oncotarget.5741

**Published:** 2015-10-15

**Authors:** Chi Huu Nguyen, Daniel Senfter, Jose Basilio, Silvio Holzner, Serena Stadler, Sigurd Krieger, Nicole Huttary, Daniela Milovanovic, Katharina Viola, Ingrid Simonitsch-Klupp, Walter Jäger, Rainer de Martin, Georg Krupitza

**Affiliations:** ^1^ Department of Clinical Pharmacy and Diagnostics, University of Vienna, Vienna, Austria; ^2^ Clinical Institute of Pathology, Medical University of Vienna, Vienna, Austria; ^3^ Department of Vascular Biology and Thrombosis Research, Center of Biomolecular Medicine and Pharmacology, Medical University of Vienna, Vienna, Austria

**Keywords:** RELA, NFKB1, MMP1, PAR1, lymph endothelial cell migration

## Abstract

RELA, RELB, CREL, NFKB1 and NFKB2, and the upstream regulators NEMO and NIK were knocked-down in lymph endothelial cells (LECs) and in MDA-MB231 breast cancer spheroids to study the contribution of NF-κB in vascular barrier breaching. Suppression of RELA, NFKB1 and NEMO inhibited “circular chemo-repellent induced defects” (CCIDs), which form when cancer cells cross the lymphatic vasculature, by ~20–30%. Suppression of RELB, NFKB2 and NIK inhibited CCIDs by only ~10–15%. In MDA-MB231 cells RELA and NFKB1 constituted MMP1 expression, which caused the activation of PAR1 in adjacent LECs. The knock-down of MMP1 in MDA-MB231 spheroids and pharmacological inhibition of PAR1 in LECs inhibited CCID formation by ~30%. Intracellular Ca^2+^ release in LECs, which was induced by recombinant MMP1, was suppressed by the PAR1 inhibitor SCH79797, thereby confirming a functional intercellular axis: RELA/NFKB1 – MMP1 (MDA-MB231) – PAR1 (LEC). Recombinant MMP1 induced PAR1-dependent phosphorylation of MLC2 and FAK in LECs, which is indicative for their activity and for directional cell migration such as observed during CCID formation. The combined knock-down of the NF-κB pathways in LECs and MDA-MB231 spheroids inhibited CCIDs significantly stronger than knock-down in either cell type alone. Also the knock-down of ICAM-1 in LECs (a NF-κB endpoint with relevance for CCID formation) and knock-down of MMP1 in MDA-MB231 augmented CCID inhibition. This evidences that in both cell types NF-κB significantly and independently contributes to tumour-mediated breaching of the lymphatic barrier. Hence, inflamed tumour tissue and/or vasculature pose an additional threat to cancer progression.

## INTRODUCTION

The colonisation of distant organs by disseminating breast cancer cells is mainly enroute the lymphatic vasculature and the extent of lymph node metastasis is a determinant of poor prognosis [1]. The port, through which breast cancer emboli transmigrate lymphatics, is formed by 12(S)-HETE that is secreted by the tumour. 12(S)-HETE inhibits VE-cadherin expression, induces ZEB1 [2] and ICAM-1 [3, 4], and causes the retraction of lymph endothelial cells (LECs) [1–7]. The retraction and the migration of LECs away from where the tumour attaches causes the formation of large cell-free areas in the lymphendothelium, so called “circular chemorepellent induced defects” (CCIDs). CCIDs give way for the entire tumour bulk to penetrate the vasculature. The CCID *in vitro* assay resembles the pathological situation in rodents and humans, which makes it a valuable tool to study mechanisms of lymph vessel breaching quantitatively and to elucidate underlying molecular mechanisms [1]. Besides 12(S)-HETE, also the NF-κB activities of LECs as well as of breast cancer cells enforce CCID formation [2, 8]. We describe that in MDA-MB231 breast cancer cells NF-κB activity constituted MMP1 expression, which in turn activated PAR1 signalling in adjacent LECs. The PAR1 signalling pathway was traced to the mobility marker MLC2. MLC2 stimulated LEC migration causing disintegrations of the lymph endothelial barrier through which breast cancer emboli can traverse. The MMP1/PAR1 inter-cellular signalling axis was shown to stimulate the intravasation of epidermoid cancer cells into the angiogenic vasculature [9]. This axis exists also in the opposite direction - originating in the stroma and ending in tumour - thereby enhancing cancer cell mobility and invasivity [10]. Upstream of MMP1, the contribution of the individual NF-κB family members to CCID formation was studied as well.

## RESULTS

### In lymph endothelial cells preferentially the canonical NF-κB pathway stimulates CCID formation

**Figure 1:**
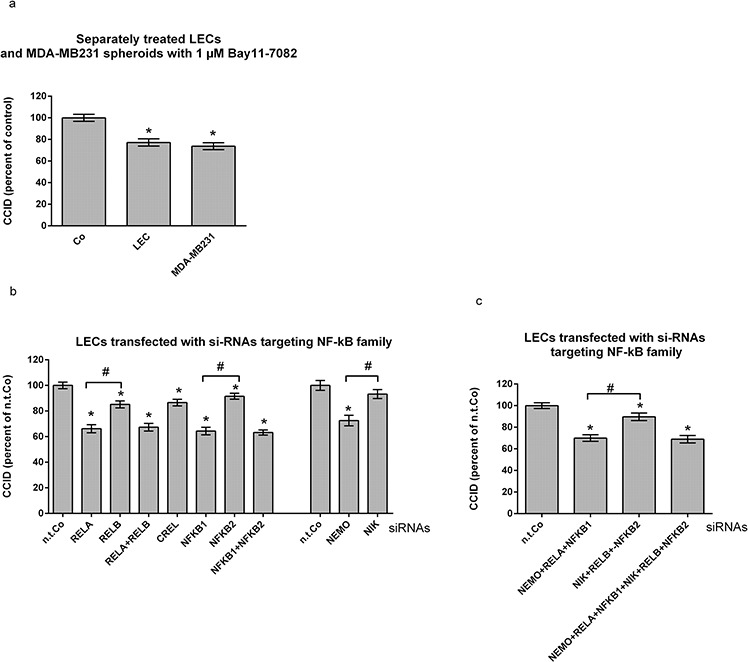
Suppression of preferentially the canonical NF-κB pathway in lymph endothelial cells (LECs) inhibits CCID formation **a.** Either LECs or MDA-MB231 spheroids were pre-treated with 1 μM Bay11–7082 for 30 min before both cell types were co-cultivated. **b, c.** LECs growing in 24-well plates to ~70–80% confluence were transiently transfected with either non-targeting (n.t.) siRNA, or siRNAs inhibiting the expression of indicated NF-κB family members, and allowed to grow to confluence. Then, MDA-MB231 spheroids were placed on top of confluent LEC monolayers and co-incubated for 4 h. The areas of CCIDs were analysed using an Axiovert microscope and Zen Little 2012 software. Experiments were performed in triplicate, error bars indicate means +/− SEM, and asterisks significance (*p* < 0.05; *t*-test).

The molecular adhesion of cancer cells to the vasculature is necessary for subsequent tumour intra-/extravasation [11] and vascular ICAM-1 significantly increases adhesion and CCID formation [3, 4]. ICAM-1 expression is induced by NF-κB and therefore, selective treatment of LECs with Bay11-7802 (irreversible IκBα inhibitor) attenuated MDA-MB231 spheroid-induced CCID formation in LEC monolayers by more than 20% (Fig. 1a). To investigate which of the NF-κB family members stimulate CCID formation the expression of RELA, RELB, CREL, NFKB1 (p50; p105), NFKB2 (p52; p100), NEMO (IκBKγ) and NIK (MAP3K14) was inhibited by transfecting respective si-RNAs into LECs (Fig. 1b). Suppression of RELA, NFKB1 and NEMO (IKBKG) attenuated CCID formation by ~ 30%, whereas suppression of RELB, CREL, NFKB2 and NIK (MAP3K14) by only ~10% or less. RELA, CREL, NFKB1 and NEMO are correlated to the “canonical” NF-κB pathway, while RELB, NFKB2 and NIK are mostly the protagonists of the “non-canonical” pathway. Therefore, siRNA combinations of canonical and non-canonical components were tested in the CCID assay but neither RELA & RELB nor NFKB1 & NFKB2 were more inhibitory than RELA alone or NFKB1 alone (Fig. 1c). Knock-down of all canonical members (RELA& NFKB1 & NEMO) had no stronger effect (~ 30%) than the separate knock-down of either component alone and this was also true for all non-canonical members (RELB & NFKB2 & NIK; ~ 10% inhibition). Knocking-down of all six components (three canonical & three non-canonical members) did not improve CCID inhibition beyond 30%. The suppression of mRNAs (SFig. 1a) and proteins was controlled by qPCR and Western blotting (SFig. 1b), respectively.

### In breast cancer cells preferentially the canonical NF-κB pathway stimulates CCID formation

**Figure 2:**
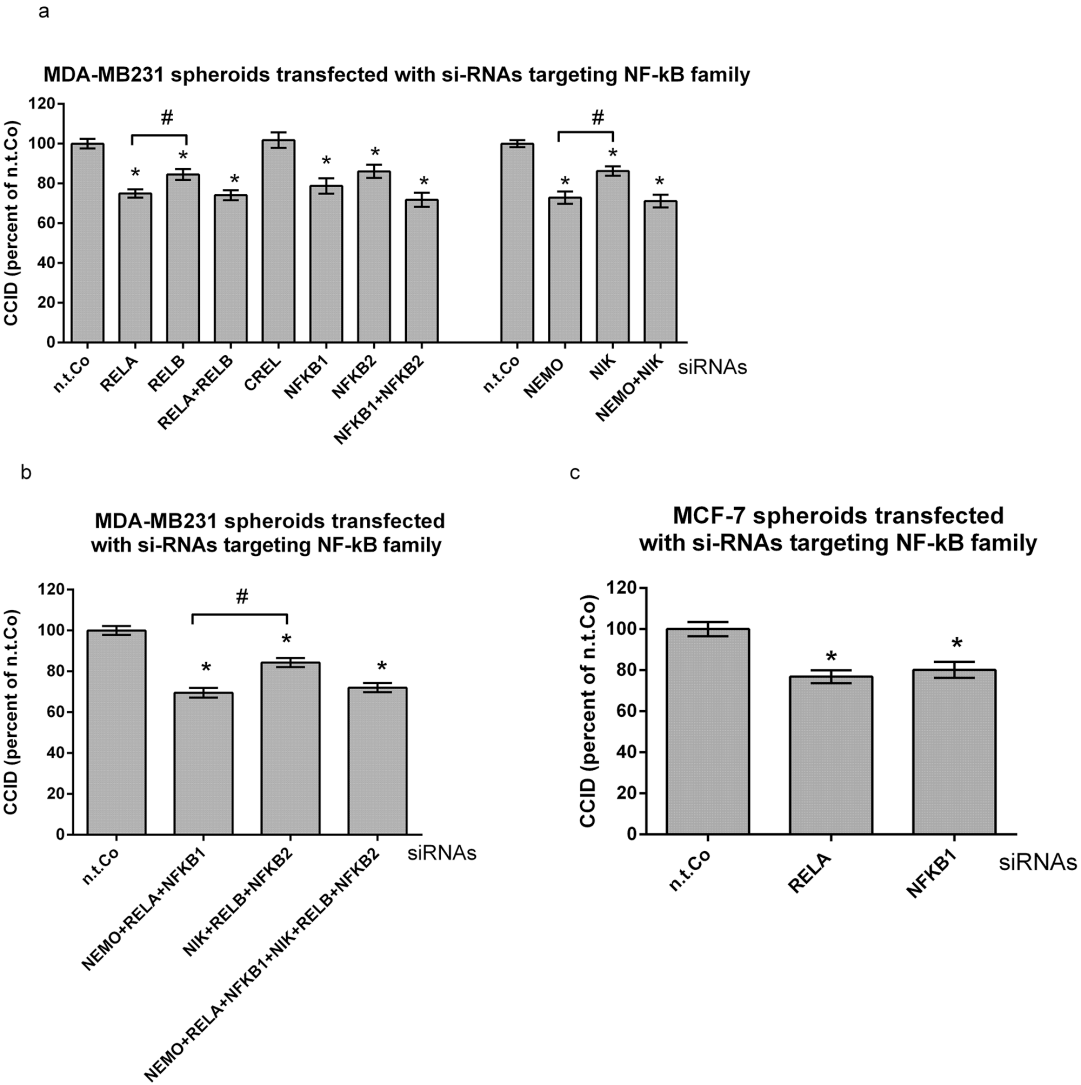
Suppression of preferentially the canonical NF-κB pathway in MDA-MB231 breast cancer spheroids inhibits CCID formation **a, b, c.** MDA-MB231- or MCF-7 spheroids were transiently transfected with either non-targeting (n.t.) siRNA, or siRNAs inhibiting the expression of indicated NF-κB family members. After 24 h, spheroids were placed on top of confluent LEC monolayers and co-incubated for 4 h. The areas of CCIDs were analysed using an Axiovert microscope and Zen Little 2012 software. Experiments were performed in triplicate, error bars indicate means +/− SEM, and asterisks significance (*p* < 0.05; *t*-test).

The selective treatment of MDA-MB231 cells with Bay11–7802 reduced CCID formation significantly (Fig. 1a). Therefore, also in MDA-MB231 cells the contribution of the NF-κB family members was studied regarding the formation of CCIDs. The knock-down of RELA, NFKB1 and NEMO in MDA-MB231 spheroids inhibited CCIDs by ~ 20–25%, whereas that of RELB, NFKB2 and NIK by only ~ 10–15% (Fig. 2a). The knock-down of CREL had no effect.

Double knock-down of RELA & RELB, NFKB1 & NFKB2, and NEMO & NIK did not inhibit CCID formation beyond single knock-down of RELA, NFKB1, or NEMO, respectively. Furthermore, the effect on CCID formation upon knock-down of either all canonical members, or of all non-canonical members, or even of all NF-κB members together (without CREL) was similar to the knock-down of just RELA or NFKB1 (Fig. 2b). Also in MCF-7 breast cancer cells RELA and NFKB1 triggered CCID formation (Fig. 2c). SiRNA-mediated suppression of respective mRNAs and proteins was analysed by qPCR and by Western blotting (SFig. 2a–2d).

### The expression of NF-κB correlates directly with that of matrix metalloproteinase 1 (MMP1)

**Figure 3:**
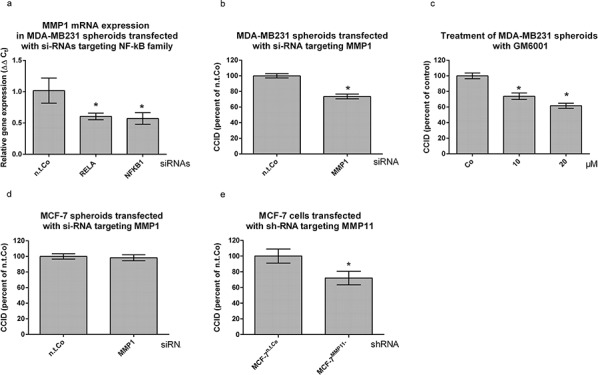
MMP1 expression is stimulated by NF-κB and contributes to CCID formation **a.** MDA-MB231 spheroids were transiently transfected with either non-targeting (n.t.) siRNA or siRNAs inhibiting the expression of RELA and NFKB1. After 24 h the mRNA was isolated, reverse transcribed to cDNA and MMP1 expression measured by qPCR (normalized to GAPDH mRNA). **b.** MDA-MB231 spheroids were transiently transfected with either non-targeting (n.t.) siRNA, or siRNA inhibiting the expression of MMP1. After 24 h, spheroids were placed on top of confluent LEC monolayers, co-incubated for 4 h, and the areas of CCIDs analyzed. **c.** MDA-MBM231 spheroids were pre-treated with 10 and 20 μM pan-MMP inhibitor GM6001 for 30 min. Then, MDA-MBM231 spheroids were placed on top of confluent LEC monolayers, co-incubated for 4 h and the areas of CCIDs were analyzed. **d.** MCF-7 spheroids were transiently transfected with either non-targeting (n.t.) siRNA, or siRNA inhibiting the expression of MMP1. After 24 h, spheroids were placed on top of confluent LEC monolayers and co-incubated for 4 h when the areas of CCIDs analyzed. **e.** MCF-7 cells transfected with either non-targeting (n.t.) control RNA, or shRNA inhibiting the expression of MMP11 were grown to spheroids, placed on top of confluent LEC monolayers and co-incubated for 4 h. The areas of CCIDs were analysed using an Axiovert microscope and Zen Little 2012 software. Experiments were performed in triplicate, error bars indicate means +/− SEM, and asterisks significance (*p* < 0.05; *t*-test).

Knock-down of RELA was shown to inhibit MMP1 mRNA expression [12-GSE30670], and also in the present investigation knock-down of RELA or NFKB1 decreased MMP1 mRNA- and protein expression in MDA-MB231 cells (Fig. 3a, SFig. 3a, respectively). Hence, the contribution of MMP1 to CCID formation was tested. The transfection of MMP1-specific siRNA (si-MMP1) into MDA-MB231 spheroids, inhibited MMP1 mRNA and protein expression (SFig. 3b, SFig. 3c, respectively) and reduced CCID formation by ~ 25% (Fig. 3b). Also the treatment of tumour spheroids with 10 μM and 20 μM of the pan-MMP inhibitor GM6001 attenuated CCID formation by 25% and 35%, respectively (Fig. 3c). In MCF-7 cells constitutive MMP1 expression was not detectable [13, 14] (SFig. 3d) and accordingly, transfection of si-MMP1 did not inhibit CCID formation in the MCF-7 model (Fig. 3d). Taking together, this implicated that in MDA-MB231 cells the CCID-stimulating effect of RELA/NFKB1 was transduced by MMP1.

Although MMP1 did not contribute to MCF-7-induced CCID formation GM6001, nevertheless, inhibits CCIDs in the MCF-7/LEC model. Instead of MMP1, MMP11 is over-expressed in MCF-7 spheroids [1] and in a variety of human carcinomas [15–17], and MMP11 expression directly correlates with improved cell-matrix adhesion [18], tumour development [19] and poor prognosis of breast cancer patients [20, 21]. Therefore, the contribution of MMP11 to CCID formation was tested. Knock-down of MMP11 in MCF-7 cells reduced mRNA and protein expression (SFig. 3e, SFig. 3f, respectively), and CCID formation by ~30% (Fig. 3e), thereby evidencing that various MMP members can contribute to the breaching of LEC barriers and may partially substitute for each other.

### Lymphendothelial Protease-Activated Receptor 1 (PAR1) transduces the MDA-MB231-delivered MMP1 signal

**Figure 4:**
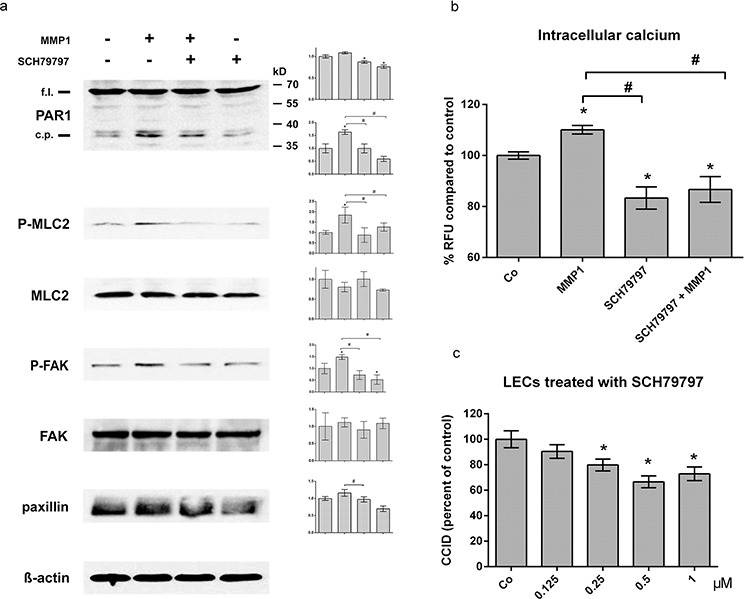
MMP1 induces Ca^2+^ signalling, and activates migratory proteins and CCIDs in LECs **a.** LECs were grown to ~70–80% confluence and then pre-treated with 0.5 μM SCH79797 or solvent (DMSO) and then stimulated with 100 ng/ml activated recombinant MMP1 for 4 h. Cells were lysed, proteins were separated by SDS gel electrophoresis and analysed by Western blotting using the indicated antibodies. Equal sample loading was controlled by Ponceau S staining and Δ-actin immunoblotting. Densitometer readings facilitated the comparison of relative protein expression levels with solvent treated control (which was set as “1”). (b) LECs (8 × 10^3^ cells/well) were pre-treated with 0.5 μM SCH79797 (PAR1 inhibitor) and then charged with FluoForte Dye-loading in presence of SCH79797 for 45 min at 37°C and 15 min at room temperature. Then, cells were stimulated with 100 ng/ml activated recombinant MMP1 for 5 min. Intracellular free calcium was measured with a fluorescence plate reader at 490/525 nm. Experiments were performed in triplicate, error bars indicate means +/− SEM, and asterisks and rhomboids significance (*p* < 0.05; *t*-test). (c) Confluent LECs were pre-treated with SCH79797 or solvent (DMSO) for 30 min and then MDA-MB231 spheroids were placed on top of LECs monolayers and co-incubated for 4 h. The areas of CCIDs were analysed using an Axiovert microscope and Zen Little 2012 software.

SDS-PAGE shows that the full length form of PAR1 migrated slightly below 70 kD, but when LECs were treated with activated MMP1 (100 ng/ml) the generation of a PAR1 cleavage product migrating at ~ 38–39 kD was stimulated. PAR1 cleavage was inhibited upon pre-treatment with the selective non-peptide PAR1 antagonist SCH79797 (Fig. 4a). The PAR1 cleavage product was reminiscent to that generated upon binding of the PAR1-activating ligand thrombin, which triggers Ca^2+^ signalling [22]. Also MMP1 treatment rapidly and transiently (SFig. 4) increased the intracellular free Ca^2+^ level in LECs (Fig. 4b), which was inhibited by SCH79797. Recombinant MMP1 significantly induced the phosphorylation of MLC2 and FAK, which is indicative for their activation, and increased the expression of paxillin (which was, however, not significant; Fig. 4a). Inhibiting PAR1 with SCH79797 caused the de-phosphorylation of MLC2 and FAK and repression of paxillin. MLC2, FAK and paxillin facilitate rapid cell movement [23–25] as it is observed during CCID formation [26]. Consistently, the inhibition of PAR1 inhibited MDA-MB231-triggered CCIDs (Fig. 4c). This evidenced that PAR1 activity contributed to LEC migration and CCID formation, which was stimulated by RELA/NFKB1 - MMP1 signals generated in MDA-MB231 breast cancer spheroids.

### Simultaneous inhibition of NF-κB in MDA-MB231 and in LECs inhibits CCID formation additively

**Figure 5:**
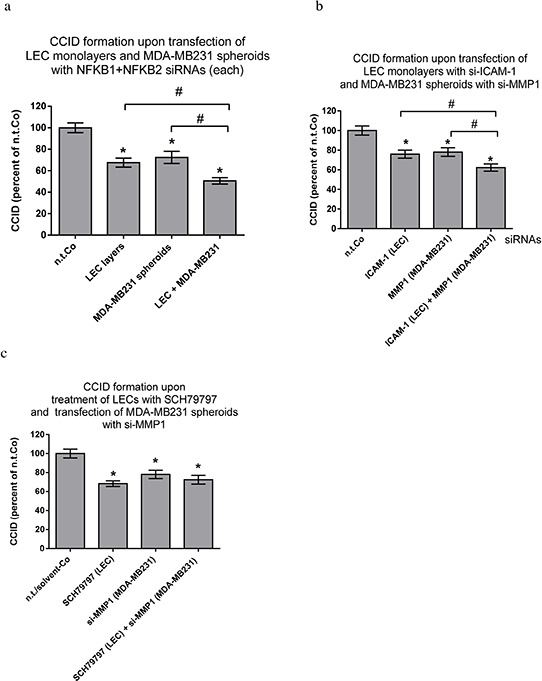
Combined inhibitions of NF-κB and target genes in MDA-MB231 spheroids and LEC monolayers LECs or MDA-MB231 spheroids were transiently transfected with either non-targeting (n.t.) siRNA or a combination of siRNAs inhibiting the expression of **a.** NFKB1 & NFKB2, **b.** or siRNAs inhibiting the expression of ICAM-1 and MMP1 respectively. After 24 h, spheroids were placed on top of confluent LEC monolayers and co-incubated for 4 h. **c.** MDA-MB231 spheroids were transiently transfected with either non-targeting (n.t.) siRNA or siRNA inhibiting the expression of MMP1. After 24 h, spheroids were placed on top of confluent LEC monolayers, which were pre-treated with 1 μM SCH79797 (inhibitor of PAR1) or solvent (DMSO) for 30 min, and co-incubated for 4 h. The areas of CCIDs were analysed using an Axiovert microscope and Zen Little 2012 software. Experiments were performed in triplicate, error bars indicate means +/− SEM, and asterisks and rhomboids significance (*p* < 0.05; *t*-test).

MDA-MB231 spheroids and LECs were transfected with a combination of si-NFKB1 & si-NFKB2, each, and the inhibition of both NF-κB pathways in both cell types inhibited CCID formation additively (Fig. 5a). This was also accomplished when the endpoints of the NF-κB pathways in MDA-MB231 and LECs were simultaneously inhibited by si-MMP1 and si-ICAM-1 [3], respectively (Fig. 5b). The suppression of ICAM-1 in LECs was analysed by Western blotting (SFig. 5). In contrast, the simultaneous knock-down of MMP1 in MDA-MB231 spheroids and of PAR1 in LECs did not result in additive inhibition of CCIDs, because MMP1 and PAR1 (unlike MMP1 and ICAM-1) are on the same trans-cellular signalling axis (Fig. 5c).

## DISCUSSION

### The stimulation of CCID formation by RELA/NFKB1

The breaching of LEC barriers by MCF-7 spheroids is stimulated by NF-κB-driven expression of ICAM-1 [3] and by NF-κB-dependent repression of VE-cadherin in LECs [2] supporting the fact that NF-κB stimulates cancer metastasis [27, 28]. Although RELB, which causes cancer progression [29, 30], significantly induced CCID formation the major role in this process was attributed to RELA/NFKB1 (“canonical” pathway). NFKB2 which is the main dimerization partner of RELB (“non-canonical” pathway) is transcribed by RELA/NFKB1 [31, 32]. Thus, the activity of RELA/NFKB1 seems to be rate-limiting. This was the likely reason why the activities of both pathways did not stimulate CCID formation beyond the activity of just RELA/NFKB1 in MDA-MB231 breast cancer cells and also in LECs. On the basis that curcumin inhibits NF-κB DNA binding, a phase II trial is currently recruiting breast cancer patients (ClinicalTrials.gov Identifier: NCT01740323) and a few other studies on curcumin are in the pipeline. Another curcumin study on lung inflammation has finished without yet showing the results (ClinicalTrials.gov Identifier: NCT01514266). There is also a multiple myeloma study testing thalidomide, which was shown to inhibit NF-κB (ClinicalTrials.gov Identifier: NCT00258245). However, in our hands thalidomide did not inhibit CCIDs when the model was treated with concentrations up to 60 μM [33]. Other clinical trials specifically focussing on the down-regulation of NF-κB activity do not exist, which is probably due to the fact that this is a very general pathway and its inhibition would cause side effects beyond tolerance. On the other hand, powerful NF-κB inhibitors are constituents of herbal remedies used in traditional medicine. Tanacetum parthenium contains the sesquiterpene lactone parthenolide and is used in plant medicine against arthritis and migraine [34]. Also a number of flavonoids, which are contained in fruits and vegetables in considerable concentrations, inhibit NF-κB activity. Still, the concentrations are apparently too low to combat cancer metastasis. For a preventive effect, however, the concentrations might do, although no studies exist confirming such positive effects in humans.

### MMP1 transduces the NF-κB signal from tumour cells to PAR1 in LECs

In MDA-MB231 cells RELA establishes constitutive expression of MMP1, which contains a functionally competent NF-κB binding site in the promoter region [13]. MMP1 is over-expressed in metastatic breast cancer and enhances invasivity [35–38]. Conversely, in MCF-10A breast hyperplasia cells, which are non-tumourigenic and do not pass through the lymph endothelial barrier, MMP1 expression is below the level of detection [39-GSE 33340, 1]. MMP1 is a marker for poor prognosis in oesophageal- and breast cancer [40, 41] and is expressed also in tumour associated stroma.

The binding of MMP1 to PAR1, such as that of the blood clotting factor Xa, activated protein C, thrombin and plasmin [10], caused PAR1 activation and subsequent stimulation of Ca^2+^ release. This, in turn, activated lymphendothelial MLC2 and FAK, which contribute to directional cell migration as a prerequisite for CCID formation. PAR1 enhances the invasivity of breast- and colon cancer as well as metastasis of melanoma, lung-, pancreas- and prostate cancer [42–47] by inducing cell migration [48] and therefore, it is considered as a proto-oncogene [49]. MCF-7 cells, which were transfected with PAR1 increased their invasive potential when orthotopically transgrafted into mammary fat pads of nude mice and accordingly, specific inhibition of PAR1 in MDA-MB231 cells attenuated invasion [10]. PAR1 was recently reported to increase intravasation of epidermoid cancer cells into the haemangiogenic vasculature [9] and here we established that PAR1 stimulated the disintegration of the lymph endothelial barrier. MDA-MB231 spheroids contacted LECs only at their apical side which can only take place at the luminal side of the lymphendothelial vasculature. Also recombinant MMP1 contacted LECs and PAR1 apically. Therefore, the here investigated mechanisms only allow conclusions regarding tumour extravasation (rather than intravasation).

Since MMP1 is located outside the cell, this makes it a prime anti-cancer target. However, from Phase III clinical trials using MMP inhibitors the observed side toxicity was ascribed to the inhibition of MMP1 [50] and therefore, the inhibition of PAR1 may be a more promising approach. PARs modulate cell response to proteolytic challenges and specifically, MMP1-PAR1 plays a significant role in arteriosclerosis [51], arterial stenosis [48] and other inflammatory conditions [52]. Noteworthy, the MMP1-PAR1 axis works in several ways: not only from tumour to lymphatics but also from stroma to tumour, because stromal MMP1 was shown to activate PAR1 in breast cancer cells thereby inducing cancer cell migration and invasivity [10].

### The contribution of MMPs and NF-κB in MCF-7-induced CCID formation

MMP1 was not detected in MCF-7 cells [13, 14] and therefore, did not contribute to CCID formation. Nevertheless, NF-κB stimulated CCID formation in the MCF-7/LEC model [2]. ICAM-1 and its counter receptor establish adhesion of MCF-7 spheroids to LEC barriers as a prerequisite for CCID formation. The counter receptor for vascular ICAM-1 [3, 4, 53] consists i.e. of the ITGAM(CD11b)/ITGB2(CD18) heterodimer. In MCF-7 cells ITGAM expression is driven by RELA [54-GSE45713]. Therefore, as a prerequisite for lymph endothelial barrier breaching the ICAM-1-dependent adhesion between LECs and breast cancer cells seems to be a rather general mechanism, which can be manipulated in various ways.

Instead of MMP1, MCF-7-triggered CCID formation was enhanced by MMP11. Also MMP2 and MMP9 were shown to contribute to endothelial barrier breaching in the MCF-7/LEC model [1]. The malignant effect of MMP2 in tumour progression is established for melanoma, lung-, prostate-, colorectal, ovarian- and breast carcinoma [55]. MMP2 and MMP9 are over-expressed in the invadopodia at the leading edge of tumours [56], but also when MMP2 is expressed in the surrounding stroma or MMP9 at pre-metastatic sites they can exert pro-metastatic activity [57, 58]. Yet, clinical trials evidence that therapies focussing on MMP2 repression are rather ineffective [59] most likely due to the high complexity of MMP functions.

Silencing MMP9 in prostate cancer cells concomitantly inhibits ICAM-1 expression [60]. Since ICAM-1 expression of LECs significantly contributes to CCID formation based on their adhesion to MCF-7 cells [3] and to neutrophils [4] this may depend not only on NF-κB [3] but also on MMP9. Taking together, tumours of even the same entity, or the stroma itself, can employ versatile mechanisms, which cause endothelial breaching.

In the MCF-7/LEC model 12(S)-HETE, which is an arachidonic acid metabolite produced by lipoxygenase ALOX15, is a major trigger of CCID formation [1, 7]. Also neutrophils depend on lymphendothelial ICAM-1 expression to adhere to their surface, stimulate CCID formation and transmigrate through the TNFα-activated lympendothelium by secreting 12(S)-HETE [4]. In MCF-7 cells the expression of ALOX15 and the production of 12(S)-HETE was independent of NF-κB activity, nevertheless, the 12(S)-HETE–induced expression of ICAM-1 in LECs could be attenuated by the specific IκBα inhibitor Bay11–7802 [2]. In the neutrophil/LEC model the expression of ICAM-1 and MMP9 was most likely linked to NF-κB activity, because it required the pre-treatment of LECs with TNFα. Whether the contribution of MMP9 to CCID formation was linked to NF-κB activity in the MCF-7/LEC model awaits its investigation.

Another mechanism modifying 12(S)-HETE-induced CCID formation is the expression and secretion of microRNA-200 (miR200) family members by CCL227 cells in a colorectal cancer (CRC)/LEC model. In particular, miR200c slowed-down the formation of CCIDs presumably due to the down-regulation of ZEB1 and other ZEB family members in LECs, which reduces their migratory potential [61]. Consistently, 12(S)-HETE, which induces LEC migration, induces also ZEB1 and this, again, depends on NF-κB activity [2]. Although 12(S)-HETE production itself does not require NF-κB activity the transduction of the signal downstream to LECs, significantly depends on NF-κB. NF-κB was shown to contribute to the up-regulation of adhesion molecules and the adhesion of tumour spheroids to LECs is rate limiting for CCID formation.

## MATERIALS AND METHODS

### Antibodies and reagents

Polyclonal rabbit anti-phospho-myosin light chain 2 (Ser19), polyclonal rabbit anti-myosin light chain 2, polyclonal rabbit anti-focal adhesion kinase (FAK) and polyclonal rabbit anti-phospho-FAK (Tyr397), NF-κB family member antibody sampler kit, polyclonal rabbit anti-NIK, and monoclonal mouse anti-IκBKγ (anti-NEMO) were from Cell Signaling (Danvers, MA, USA). Polyclonal rabbit anti-RELA (p65), polyclonal rabbit anti-NFKB1 (p50) were ordered from Santa Cruz Biotechnology (Heidelberg, Germany), polyclonal rabbit anti-MMP1 was from Abcam (Cambridge, UK) and mouse monoclonal anti MMP11 from Chemicon International (Temecula, CA, USA). Polyclonal goat anti-CD54 (ICAM-1) and monoclonal mouse anti-human PAR1 was from R&D system (Minneapolis, MN, USA). Monoclonal mouse anti-β-actin was from Sigma-Aldrich (Munich, Germany). Polyclonal rabbit anti-mouse, polyclonal swine anti-rabbit and polyclonal rabbit anti goat IgGs were purchased from Dako (Glostrup, Denmark).

siRNAs targeting IκBKγ (NEMO; ID# s16186, cat no.: 4390824), RELA (ID# s11914, cat.no.: 4390824), ICAM-1 (ID# s7087, cat.no.: 4392420), and non targeting (n.t.) control siRNA (Silencer Select Negative Control No. 1 siRNA, cat.no.: 4390843) were from Ambion (Life Technologies, Carlsbad, CA, USA). siRNAs targeting human MAP3K14 (NIK; SMART pool “ON-TARGET plus”, cat.no.: L-003580–000005), RELB (cat.no.: L-004767–00-0005), CREL (cat.no.: L-004768–00-0005), NFKB1 (cat.no.: L-003520–00-0005), NFKB2 (cat.no.: L-003918–00-0005) and MMP1 (cat.no.: L-005951–00-0005) were ordered from Dharmacon (Gene Expression and Gene Editing, GE Healthcare, Lafayette, CO, USA).

All siRNAs were re-suspended in RNAse-free water to make a stock concentration of 20 μM. Increasing the amount of non targeting (n.t.) control RNA did not change the outcome on CCID formation (data not shown).

The IκBα phosphorylation inhibitor (E)-3-[(4-methylphenylsulfonyl]-2-propenenitrile (Bay11–7082) was purchased from Calbiochem (Darmstadt, Germany). SCH79797 hydrochloride was obtained from Axon Medchem (Groningen, Netherlands), human recombinant MMP1 from Sigma-Aldrich (SRP3117, MO, USA).

### Cell culture

Human MDA-MB231 and MCF7 breast cancer cells were purchased from the American Type Culture Collection (ATCC, Rockville, MD, USA) and grown in MEM medium supplemented with 10% foetal calf serum (FCS), 1% penicillin/streptomycin (PS) and 1% non-essential amino acids (Gibco, Invitrogen, Karlsruhe, Germany). Telomerase immortalized human lymph endothelial cells (LECs) were grown in EGM2 MV (Clonetics CC-4147, Allendale, NJ, USA). The cells were kept at 37°C in a humidified atmosphere containing 5% CO_2_. For CCID formation assays, LECs were stained with cytotracker green purchased from Invitrogen (Karlsruhe, Germany).

### Spheroids formation

MDA-MB231 cells (input of 6.000 cells per spheroid; when used in experiments the average spheroid diameter was ~ 338 μm overcasting a LEC area of ~ 104000 μm^2^) and MCF-7 cells (input of 3000 cells/spheroid; when used in experiments the average spheroid diameter was ~463 μm overcasting a LEC area of ~164000 μm^2^) were transferred to 30 ml serum free MEM medium containing 6 ml of a 1.6% methylcellulose solution (0.3% final concentration). 150 μl of this cell suspension were transferred to each well of a 96-well plate (Greiner Bio-one, Cellstar 650185, Kremsmünster, Austria) to allow spheroid formation within 72 h.

### CCID (circular chemorepellent induced defect) assay

In this assay the sizes of the cell free areas (circular chemorepellent induced defects; CCIDs), which are formed in the endothelial monolayer directly underneath the tumour spheroids, were measured [1]. MDA-MB231spheroids were washed in PBS and transferred to cytotracker-stained LEC monolayers that were seeded into 24-well plates (Costar 3524, Sigma-Aldrich, Munich, Germany) in 1 ml EGM2 MV medium. After 4 h of incubation, the CCID areas in the LEC monolayers underneath the MDA-MB231 spheroids were photographed using an Axiovert (Zeiss, Jena, Germany) fluorescence microscope to visualise cytotracker(green)-stained LECs underneath the spheroids. CCID areas were calculated with the Zen Little 2012 (Zeiss, Jena, Germany). For each condition the CCID size of 15 or more spheroids (unless otherwise specified) was measured.

### Transfection of LEC monolayer

LECs were seeded in 24-well plate (1 ml/well) and grown in EGM2 medium. Transfections were performed when the cells had a confluence of 70–80%. A total of 0.75 μg siRNA (3 μl from 20 μM stock) and 6 μl Hiperfect Transfection Reagent (Qiagen, cat. no.: 301705) were mixed in 100 μl serum-free medium and incubated for 30 min at room temperature to allow the formation of transfection complexes. The old cell culture medium was gently removed and 500 μl of fresh EGM2 medium were added into each well. Then the transfection complexes were added drop-wise to the cells (to a final siRNA concentration of 100 nM) and incubated for 24 h at 37°C. After 24 h, the medium was replaced by fresh medium and cells were incubated for another 24 h to recover. The LECs monolayers were used for CCID assays or isolated RNA for qPCR.

### Transfection of MDA-MB231 spheroids

On the day of transfection, spheroids were washed with PBS and transferred to 15 ml tubes containing 500 μl serum-free medium. MDA-MB231 spheroids were transfected with different siRNAs using Hiperfect Transfection Reagent as mentioned above. After 24 h, spheroids were used for CCID assays or isolated RNA for qPCR.

### Lentiviral transduction of shMMP11 into MCF-7 cells

MCF-7 cells were grown in 24 well plates to 80% confluence in MEM medium supplemented with 10% FCS, 1% penicillin/streptomycin, 1% NEAA and 150 μg hygromycin B. To increase the efficiency of the transduction and to prevent repulsion between cancer cells and virus particles (Mission lentiviral transduction particles; Cat. No. SHVRS; five clones, IDs: TRCN0000050713, TRCN0000050714, TRCN0000050715, TRCN0000050716, TRCN0000050717) both, cancer cells and virus particles were treated with polybrene (hexadimethrine bromide final concentration 8 μg/ml; Cat. No. H9268 from Sigma-Aldrich (Munich, Germany), and the cell-viral particle mixture was centrifuged at 2000 g for 90 min at 32°C (spin infection). After 24 h of incubation at 37°C the cells were washed with PBS, trypsinised, and re-seeded under the above mentioned culture conditions (without hygromycin B). The next day and then every 3–4 days the medium was replaced with fresh, puromycin (1 μg/ml) containing medium, until resistant colonies emerged, which were individually analysed.

### Quantitative RT-PCR (qPCR)

Cells or spheroids were harvested after transfection and RNA was isolated using the RNeasy Mini Kit 50 and QIAshredder 50 (QIAGEN, Hamburg, Germany). The final RNA concentration was measured using a NanoDrop Fluorospectrometer (Thermo Fisher Scientific, Inc., Waltham, MA, USA). An amount of 2 μg of total RNA was reverse transcribed using RNA to cDNA EcoDry Premix Protocol-At-A-Glance (Clontech, 2 Saint-Germain-en-Laye, France), the resulting cDNA was amplified using TaqMan Gene Expression Master Mix (Applied Biosystems, Vienna, Austria). The PCR products were analyzed on the Chromo4 PCR System (Bio-Rad, Hercules, CA, USA). The following TaqMan probes were used: GAPDH (Hs99999905_m1), IKBKG (NEMO; Hs00415849_m1), MAP3K14 (NIK; Hs00177695_m1), RELA (Hs00153294_m1), RELB (Hs00232399_m1), CREL (Hs00968440_m1), NFKB1 (p100; Hs00765730_m1), NFKB2 (p105; Hs00174517_m1), and MMP1(Hs00899658_m1). qPCR was performed in triplicate for each cDNA template. Gene expression normalized to GAPDH expression (glyceralaldehyde 3-phosphate dehydrogenase) and was calculated using the ΔΔC_T_ method.

### SDS gel electrophoresis and Western blotting

LECs were growth in T-25 tissue culture flasks (Nunc, Roskilde, Denmark) to 80% confluence and pre-treated with 0.5 μM SCH79797 hydrochloride for 1 h and then stimulated with 100 ng/ml MMP1 for another 4 h. Afterwards, cells were washed twice with ice cold PBS and lysed in buffer containing 50 mM Tris-HCl (ph = 6.8), 6% SDS, 20% glycerin, 1.85 mM EDTA, phosphatase inhibitor cocktail and protease inhibitor cocktail. For complete cell lysis, the mixture was sonicated 5–10 times on ice. The lysate was stored at −20°C until further analysis. Equal amounts of protein were separated by SDS polyacrylamide gel electrophoresis and electro-transferred onto Amersham Hybond-P PVDF transfer membrane (GE Healthcare, Freiburg, Germany) at 100 V for 1 h in cold transfer buffer (containing 20 mM Tris-base, 150 mM glycine, 20% (v/v) methanol, pH 8.5). Membranes were stained with Ponceau S (Sigma-Aldrich, Munich, Germany) to control transfer efficiency and equal sample loading. After washing with TBS/T (Tris Buffered Saline/Tween 20; pH 7.6), membranes were immersed in blocking solution (5% non-fat dry milk in TBS containing 0.1% Tween) at room temperature for 1 h. Membranes were washed and incubated with primary antibodies (in blocking solution; dilution 1:500 – 1:1000) by gently rocking at 4°C overnight. Thereafter, the membranes were washed with TBS/T and incubated with secondary antibodies (peroxidase-conjugated swine anti-rabbit IgG; dilution 1:5000 or rabbit anti-mouse, rabbit anti-goat IgG, dilution 1:10000) at room temperature for 1 h. Chemo-luminescence was developed by Amersham ECL prime Kit (GE Healthcare, Freiburg, Germany) and detected using a Lumi-Imager F1 Workstation (Roche, Basel, Switzerland). Densitometry of the Western blots was analysed with Image-J software (National Institutes of Health, Maryland, USA).

### Intracellular Ca^2+^ assay

Free intracellular Ca^2+^ levels were measured using FluoForte Calcium Assay Kit (Enzo Life Sciences, Ann Arbor, MI, USA). 8 × 10^3^ LECs/well/100 μl EGM2 medium were seeded into 96-well black-wall clear-bottom plates (Nunc, Thermo scientific, NY, USA) and after 24 h LECs were pre-treated with 0.5 μM SCH79797 for 30 min. Then, medium was removed and 100 μl FluoForte Dye-loading (containing SCH79797 where applicable) was added to each well. Cells were further incubated for 45 min at 37°C followed by 15 min at room temperature. Then, cells were stimulated with 100 ng/ml MMP1 for 5 min and the fluorescence was measured with a fluorescence plate reader at 490/525 nm.

### Statistical analysis

For statistical analyses Excel 2013 software and Prism 6 software package (GraphPad, San Diego, CA, USA) were used. The values were expressed as mean ± SEM and the Student's *t*-test was applied to compare differences between control samples and treatment groups. Statistical significance level was set to *p* < 0.05.

## CONCLUSION

This work demonstrates the malign nature of the NF-κB-dependent MMP1-PAR1 axis in a new *in vitro* model resembling the extravasation of breast cancer emboli out of the lymphatic lumen. This links inflammation that may occur in proximity of tumour burden to early stages of metastasis.

## SUPPLEMENTARY FIGURES


