# Supplementary material for: NF-κB contributes to MMP1 expression in breast cancer spheroids causing paracrine PAR1 activation and disintegrations in the lymph endothelial barrier in vitro
Source: Oncotarget. 2015 Oct 15;6(36):39262–75. doi: 10.18632/oncotarget.5741 (PMC4770771; doi:10.18632/oncotarget.5741)
Supplement: Supplementary file 1 [file oncotarget-06-39262-s001.pdf]

SUPPLEMENTARY FIGURES

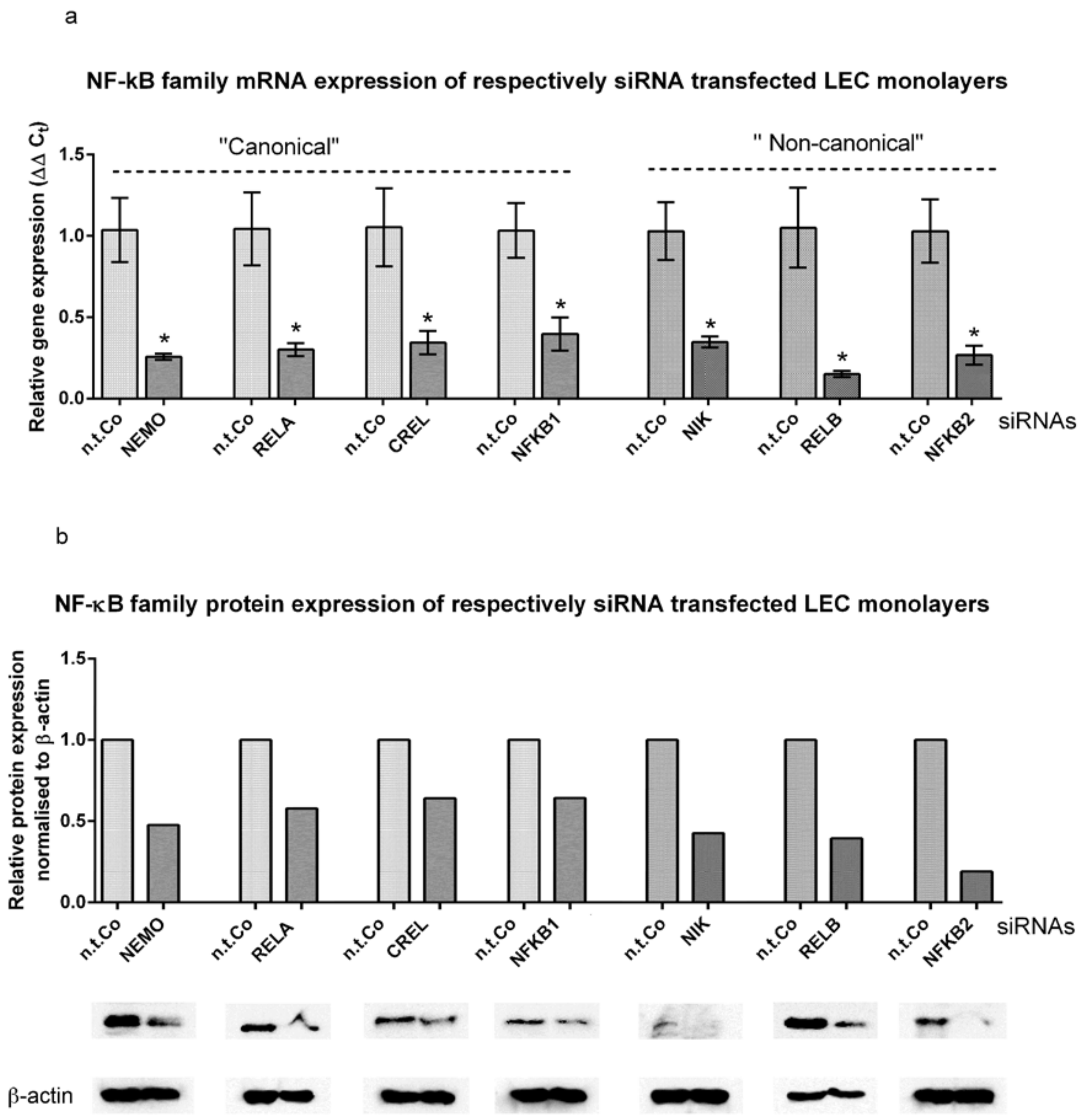

Supplementary Figure S1: Inhibition of (a) mRNA and (b) protein expression of NF-κB members in LECs by specific siRNAs. LECs growing in 24-well plates to ~70–80% confluence were transiently transfected with either non-targeting (n.t) siRNA or siRNA inhibiting the expression of indicated NF-κB members. **a.** After 24 h the mRNA was isolated, reverse transcribed and then qPCR was performed. The expression levels of RELA, RELB, CREL, NFKB1, NFKB2, NEMO and NIK were normalised to GAPDH. qPCR was performed in triplicate for each cDNA template, error bars indicate means +/− SEM, and asterisks significance ($p < 0.05$; *t*-test). **b.** After 24 h cells were lysed, total protein isolated, separated by SDS-PAGE and transferred to PVDF membranes for Western blotting using the indicated antibodies. ß-actin was used to control equal sample loading. Densitometer readings facilitated the comparison of relative protein expression levels with solvent treated control (which was set as "1").

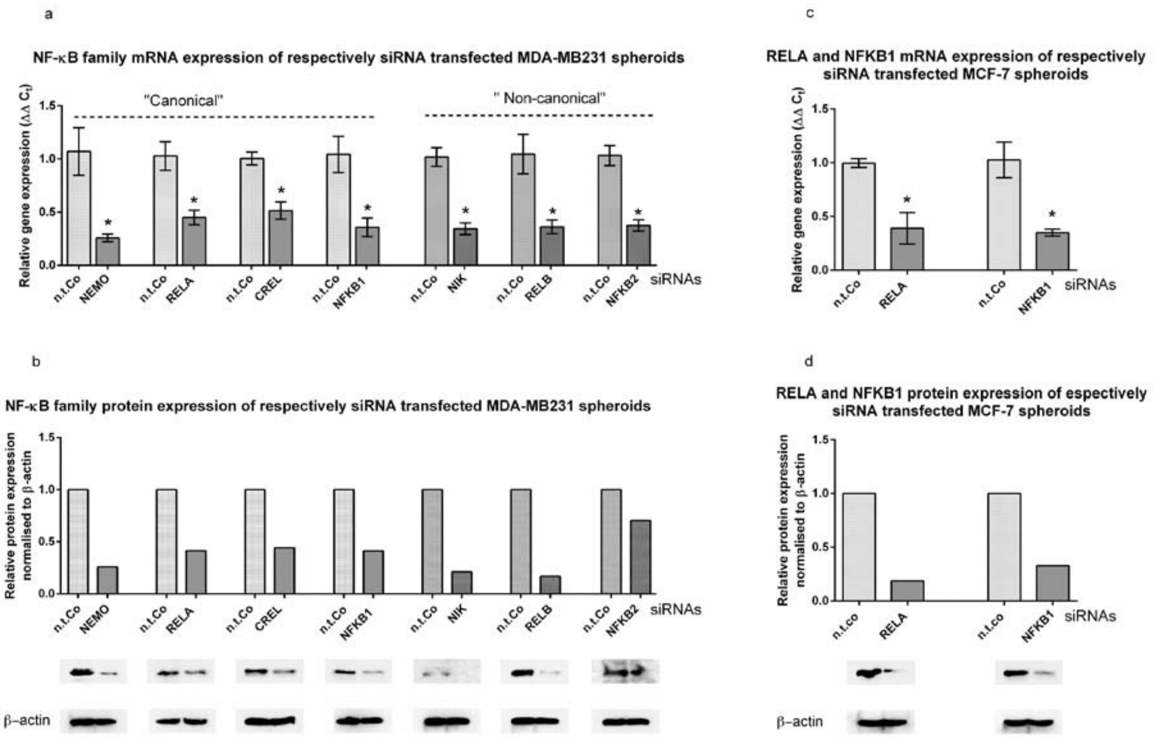

Supplementary Figure S2: Inhibition of (a) mRNA and (b) protein expression of NF-κB members in MDA-MB231 spheroids by specific siRNAs. MDA-MB231 spheroids were transiently transfected with either non-targeting (n.t.) siRNA or siRNA inhibiting the expression of indicated NF-κB members. **a.** After 24 h, the mRNA was isolated, reverse transcribed and then qPCR was performed. The expression levels of RELA, RELB, CREL, NFKB1, NFKB2, IKBKG (NEMO) and MAP3K14 (NIK) were normalised to GAPDH. qPCR was performed in triplicate for each cDNA template, error bars indicate means +/− SEM, and asterisks significance ($p < 0.05$; *t*-test). **b.** After 24 h cells were lysed, total protein isolated, separated by SDS-PAGE and transferred to PVDF membranes for Western blotting using the indicated antibodies. ß-actin was used to control equal sample loading. Densitometer readings facilitated the comparison of relative protein expression levels with solvent treated control (which was set as “1”). (c) Inhibition of RELA and NFKB1 mRNA and (d) protein expression in MCF-7 spheroids by specific siRNAs MCF7 spheroids were transiently transfected with either non-targeting (n.t.) siRNA or siRNA inhibiting the expression of RELA and NFKB1. **c.** After 24 h, the mRNA was isolated, reverse transcribed and then qPCR was performed. The expression levels of RELA and NFKB1were normalised to GAPDH. qPCR was performed in triplicate for each cDNA template, error bars indicate means +/− SEM, and asterisks significance ($p < 0.05$; *t*-test). **d.** After 24 h cells were lysed, total protein isolated, separated by SDS-PAGE and transferred to PVDF membranes for Western blotting using the indicated antibodies. ß-actin was used to control equal sample loading. Densitometer readings facilitated the comparison of relative protein expression levels with solvent treated control (which was set as “1”).

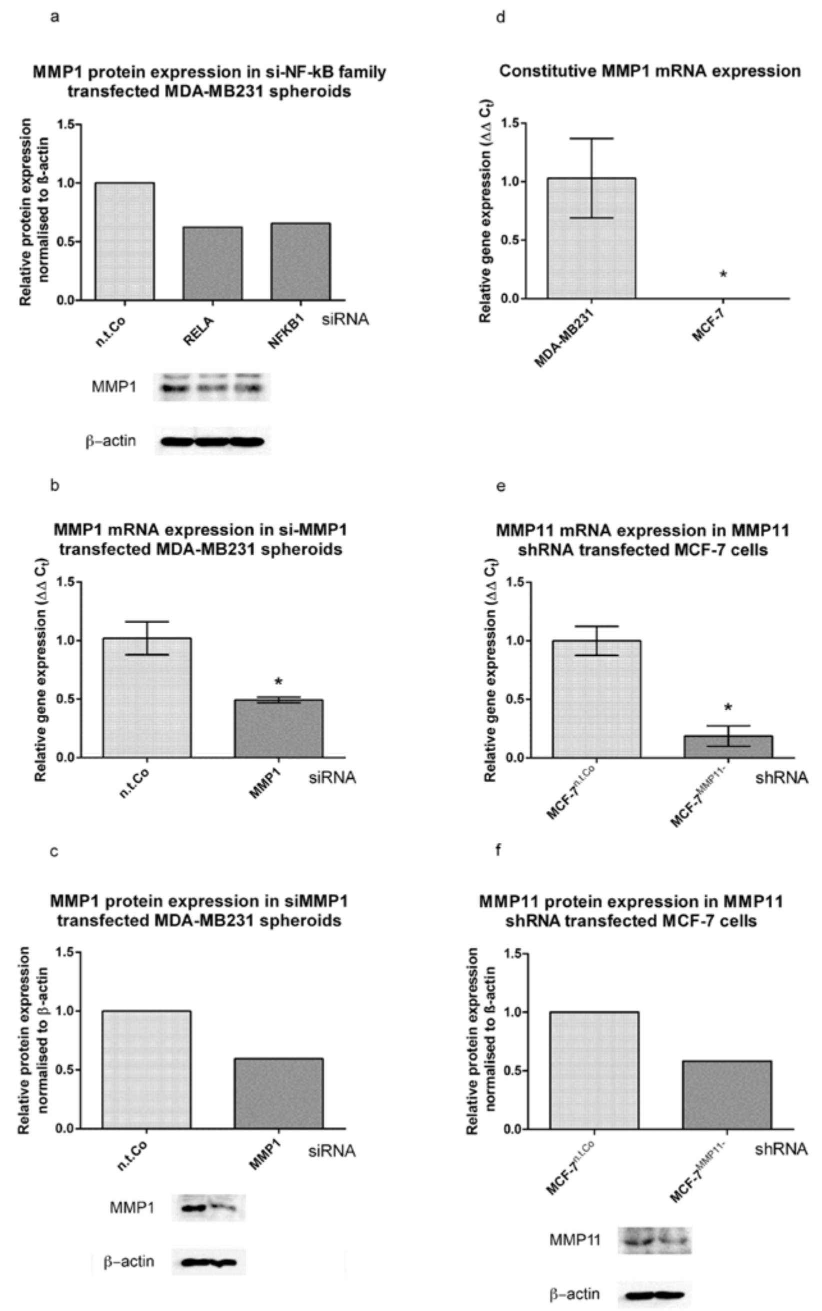

Supplementary Figure S3: Inhibition of MMP1 mRNA and protein expression in MDA-MB231 spheroids by specific siRNAs. MDA-MB231 spheroids were transiently transfected with either non-targeting (n.t.) siRNA or siRNAs inhibiting the expression of (a) RELA, NFKB1 and (b, c) MMP1. **a, c.** After 24 h cells were lysed, total protein isolated, separated by SDS-PAGE and transferred to PVDF membranes for Western blotting using the indicated antibodies. ß-actin was used to control equal sample loading. Densitometer readings facilitated the comparison of relative protein expression levels with solvent treated control (which was set as "1"). **b.** After 24 h, the mRNA was purified, reverse transcribed and then qPCR was performed. The expression levels of MMP1 were normalised to GAPDH. qPCR was performed in triplicate for each cDNA template, error bars indicate means +/− SEM, and asterisks significance ($p < 0.05$; *t*-test). **d.** Comparative expression of MMP1 in breast cancer cells RNA was isolated from MDA-MB231 and MCF-7 cells (cells growing to confluence of ~70–80% in T-25 tissue culture flasks), reverse transcribed and then, qPCR was performed to measure MMP1 expression levels (normalized to GAPDH mRNA). **e.** Inhibition of MMP11 expression in MCF-7MMP11- cells by specific shRNA MCF-7 cells were stably transfected with non-targeting control RNA (n.t.) or shRNA inhibiting the expression of MMP11. After selection of single clones the mRNA was purified, reverse transcribed and then qPCR was performed. The expression level of MMP11 was normalised to GAPDH. qPCR was performed in triplicate for each cDNA template, error bars indicate means +/− SEM, and asterisks significance ($p < 0.05$; *t*-test). The clone, in which MMP11 was suppressed most efficiently is shown and was used for further experiments. **f.** After selection cells were lysed, total protein isolated, separated by SDS-PAGE and transferred to PVDF membranes for Western blotting using the indicated antibodies. ß-actin was used to control equal sample loading. Densitometer readings facilitated the comparison of relative protein expression levels with solvent treated control (which was set as "1").

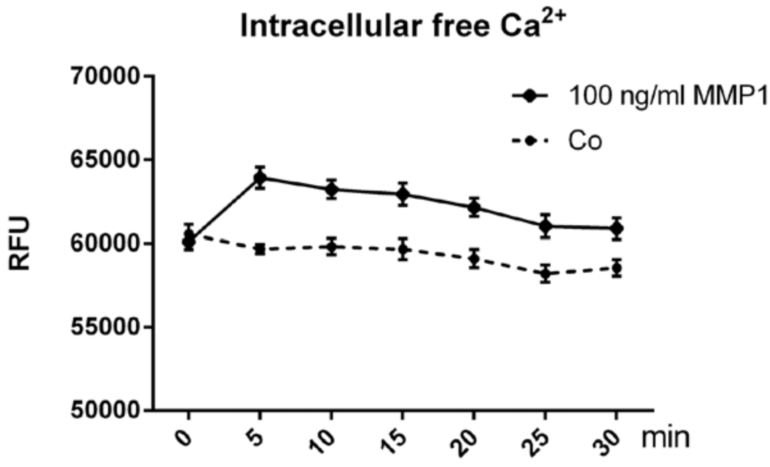

Supplementary Figure S4: MMP1 induced intracellular calcium release. LECs were incubated with FluoForte Dye-loading for 45 min at 37°C and 15 min at room temperature. Then, cells were stimulated with 100 ng/ml activated recombinant MMP1. Intracellular calcium release was measured at different time points with a fluorescence plate reader at 490/525nm.

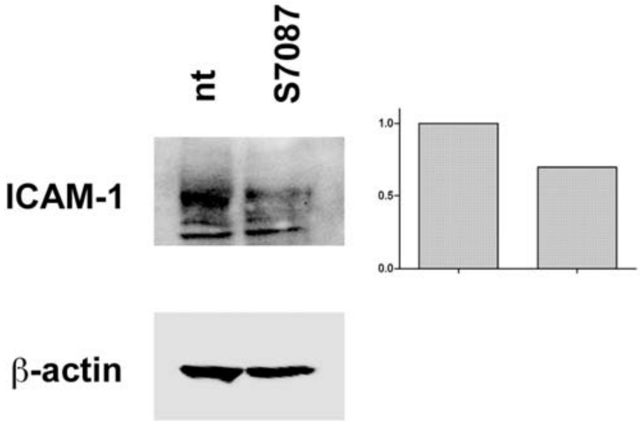

Supplementary Figure S5: Inhibition of ICAM-1 expression in LECs by specific siRNA. LECs were grown to confluence in 6-well plates and transiently transfected with either non-targeting (nt) siRNA or siRNA inhibiting the expression of ICAM-1 (S7087). After 24 h cells were lysed, proteins separated by SDS gel electrophoresis and analysed by Western blotting using indicated antibody. Equal sample loading was controlled by Ponceau S staining and ß-actin immunoblotting. Densitometer readings facilitated the comparison of relative protein expression levels with non-targeting control (nt; which was set as “1”).
